# Maintaining Traditions: A Qualitative Study of Early Childhood Caries Risk and Protective Factors in an Indigenous Community

**DOI:** 10.3390/ijerph14080907

**Published:** 2017-08-11

**Authors:** Ana Levin, Karen Sokal-Gutierrez, Anita Hargrave, Elizabeth Funsch, Kristin S. Hoeft

**Affiliations:** 1 School of Medicine, University of California, San Francisco, CA 94101, USA; Anita.Hargrave@ucsf.edu; 2 School of Public Health, University of California, Berkeley, Berkeley, CA 94720, USA; ksokalg@berekeley.edu; 3 Department of Biostatistics and Epidemiology, Zuckerman College of Public Health, University of Arizona, Tucson, AZ 85721, USA; efunsch@gmail.com; 4 Department of Preventive and Restorative Dental Sciences, University of California, San Francisco, CA 94118, USA; Kristin.Hoeft@ucsf.edu

**Keywords:** caries, nutrition transition, early childhood caries, Ecuador, Positive Deviance

## Abstract

In lower middle-income economies (LMIE), the nutrition transition from traditional diets to sugary foods and beverages has contributed to widespread early childhood dental caries. This qualitative study explores perceived risk and protective factors, and overall experiences of early childhood nutrition and oral health in indigenous Ecuadorian families participating in a community-based oral health and nutrition intervention. Dental exams of 698 children age 6 months through 6 years determined each child’s caries burden. A convenience sample of 18 “outlier” families was identified: *low-caries* children with ≤2 carious teeth vs. *high-caries* children with ≥10 carious teeth. Semi-structured in-depth interviews with parents/caregivers explored the child’s diet, dental habits, and family factors related to nutrition and oral health. Interviews were transcribed and thematically analyzed using grounded theory. In the high-caries families, proximity to highway and stores, consumption of processed-food, and low parental monitoring of child behavior were identified as risk factors for ECC (early childhood caries). In the low-caries families, protective factors included harvesting and consuming food from the family farm, remote geography, and greater parental monitoring of child behavior. The study results suggest that maintaining traditional family farms and authoritative parenting to avoid processed foods/drinks and ensure tooth brushing could improve early childhood nutrition and oral health.

## 1. Introduction

Over recent decades, lower middle income economies (LMIE) have experienced a nutrition transition from traditional diets to sugary, highly processed foods and beverages, resulting in a dramatically increased prevalence of diet-related chronic diseases, including tooth decay, obesity, type 2 diabetes, and cardiovascular disease [1,2]. Childhood tooth decay, or caries, is the most prevalent chronic disease worldwide, affecting 60–90% of children [2]. The consequences include chronic mouth pain, malnutrition, poor self-image, and diminished educational and economic potential [2,3,4]. Tooth decay results from the metabolization of carbohydrates by cariogenic oral bacteria, producing acid, which demineralizes the tooth, eventually causing cavities [3,5,6,7]. Childhood caries are often quantified by the number of decayed, missing, or filled deciduous teeth (DMFT) in children under six years of age [4,8].

Different models have characterized the risk factors and protective factors for caries development. The Keyes Caries Triad that highlights the interplay between dietary sugar consumption, oral cariogenic bacteria, and underlying health factors of the host and teeth [5]. The Caries Balance Model stresses the balance between pathological caries-producing factors (e.g., dietary sugars, acid-producing oral bacteria) with protective caries-preventing factors (e.g., salivary flow, fluoride, and antibacterial treatments) [5,6,7]. A social ecological approach encompasses factors from the microbiological, child, family and community levels, and recognizes caries as a multifactorial and multi-level disease [9]. Similar models couch this multi-level framework within the context of social and economic inequalities to better understand and address oral health disparities. 

Historically, caries prevention has targeted the individual child by promoting tooth brushing and fluoride supplementation, and later the parent/caregiver as someone who oversees the child’s diet and oral hygiene practices [10,11]. More recent interventions have addressed community-level factors, including in high-income and middle-income economies, where the widespread use of community water fluoridation, fluoride toothpaste and fluoride varnish have helped to decrease childhood caries incidence [9,12]. The World Health Organization’s *Oral Health Report* has called for wider development of policies and community-based oral health promotion particularly for poor and disadvantaged populations [9,12,13,14]. Some public health interventions have incorporated community-level policy changes such as limiting cariogenic food and beverage sales in schools, taxing sugar-sweetened foods and beverages [14], and increasing access to preventive dental care in primary health care offices and dental clinics [15,16].

Despite the increasing research focus on health disparities and marginalized populations, rural indigenous populations remain relatively underrepresented in the literature. This subgroup often faces particular challenges to oral health and nutrition from increasing urbanization and dietary transition, as well as geographic, language and cultural barriers to health care access. Very little is known about the oral health status of the Amazonian Kichwa, both past and present [14,17]. The traditional Kichwa diet consisted primarily of subsistence-farmed fruits, vegetables and nuts, occasional fish and meat, and beverages like *chicha* (a fermented drink made from manioc or corn) [18,19]. While some research suggests that *chicha* could have caused tooth decay among pre-colonial populations [20], studies carried out in the 1960s to examine the oral health of indigenous communities in the Brazilian Amazon suggest that tooth decay was rare [20]. Decades later, subsequent investigations of these Brazilian Amazonian populations demonstrate that the increase in access to sugary foods and beverages through the 1970s and 1980s led to a nutrition transition and widespread early-childhood caries [21]. Currently, Ecuador has the second highest prevalence of childhood caries in Latin America, and caries prevalence in indigenous 6-year-old children is greater than 90%, the highest in the nation [22].

Little research has addressed the family or community-level factors that promote and prevent early childhood caries in rural indigenous populations. This study aims to identify the risk factors and protective factors for nutrition and oral health among Kichwa families participating in a community-based oral health and nutrition intervention.

## 2. Materials and Methods

### 2.1. Study Design

This is a qualitative study investigating the perceived risk factors and protective factors for ECC in an indigenous Ecuadorian population. This study is nested within a larger community-based oral disease assessment and intervention to improve early childhood nutrition and oral health. Dental exams, fluoride application, and nutritional and oral health education were administered by community health volunteers and teachers [19,23]. Toothbrushes, fluoride toothpaste, fluoride varnish, and referral to local dental treatment was provided as needed [23]. The study was approved by the University of California, Berkeley Committee for the Protection of Human Subjects, with additional permission from the Chief of the local community network and Ecuadorian provincial Ministry of Health officials (2011-04-3178/0006252).

### 2.2. Study Population

The study participants were parents/caregivers of children age 6 months through 6 years living within indigenous communities in Pueblo Kichwa de Rukullakta, in the Ecuadorian Amazonian jungle region. Study participants were purposively sampled from a larger study, the aim of which was to include all children in this age group living within Pueblo Kichwa. Pueblo Kichwa is a network of 17 communities, each with its own school. Although all of the communities would be considered rural, they include a range of geography, proximity or remoteness from the highway, and access to farmland, stores and medical/dental care.

Among the participating communities involved in the larger study and the 698 child participants in 2013, there was a wide range of caries prevalence and severity by DMFT [23]. The four communities with the highest ECC prevalence were distinguished as communities with “extreme caries prevalence”, and the five communities with the lowest ECC prevalence as “very low caries prevalence”. Within each of these nine communities, individual children were identified as “outliers”—having either very high or very low numbers of decayed teeth on dental examination compared to the larger study population. The study of outliers, frequently termed “positive/negative deviance”, is an anthropological methodology first defined in the 1970s to study undernourished populations where individuals within a marginalized subgroup were observed to deviate from the general nutritional status of the subgroup [23].

**Figure 1:**
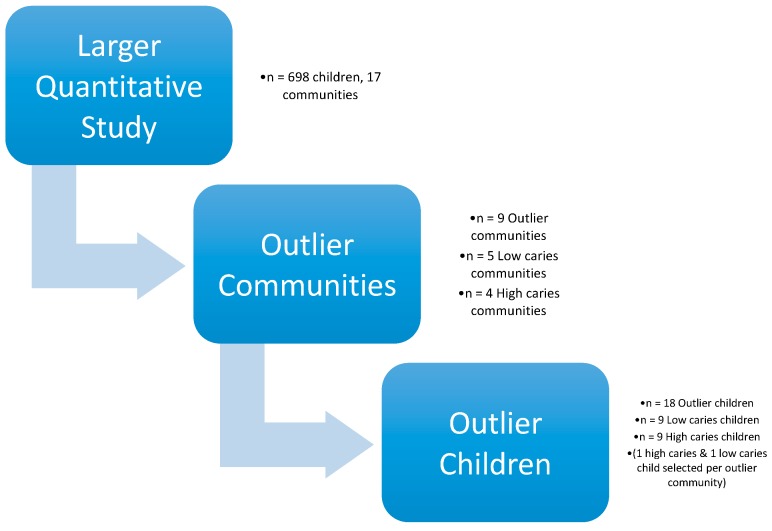
Depiction of study design.

Each community began participating in the larger study at the same time, and continued for the duration of the intervention [24]. From 2011 to 2013, over 1500 children participated in the larger intervention [23]. Children with 10 or more decayed teeth (i.e., ½ of their deciduous teeth) were categorized into the “high-caries” group, and children with 2 or fewer decayed teeth into the “low-caries” group. From each of the nine communities, one high-caries child and one low-caries child were identified by scheduling convenience, yielding a total of nine high-caries children and nine low-caries children. Outlier families were selected for study based on the existence of at least one outlier child in the household. Within each family, the child with the most extreme caries status (low or high) among their siblings was selected for the low caries group or high caries group (Figure 1).

### 2.3. Data Collection

A Kichwa-speaking health volunteer and a Spanish-speaking researcher (A.L., A.H. or E.F.) visited each family’s home, explained the study in Kichwa and/or Spanish, and received signed informed consent from the parent, usually mother, or at times both the mother and father together. In the family’s preferred language, the team conducted a semi-structured in-depth interview with each parent/caregiver regarding the child’s daily diet and oral health habits, and obstacles and facilitators for achieving good oral health and nutrition. Examples of the type of questions asked include: “Tell me about what your child usually eats and drinks in a day”, “How is your child’s diet similar or different to what you ate as a kid?”, and “What do you do to take care of your child’s teeth?”.

Interviews were audio recorded and audio files were transcribed into Spanish-language Microsoft Word documents by the research team and a hired transcriptionist. After completing several interviews, the research team listened to the audio files, transcribed the interviews, and used grounded-theory to modify and improve the interview guide [23].

### 2.4. Data Analysis

Initial data analysis involved hand-coding using broad topics of health, nutrition, socio-economic status, and education. The health and nutrition topics were selected for in-depth analysis. Using Dedoose, a qualitative analysis computer software program [25], the health and nutrition topics were coded into 3 larger codes, child, family and community. Within each of these, 50 smaller subcodes were described. Some of the subcodes identified included store ownership, land ownership, *chacra*, parental health education, parental health knowledge, and mouth pain. The most common themes were identified, defined as risk vs. protective factors, and transcripts were compared between high-caries vs. low-caries families. Finally, the specific themes were analyzed as relating to Child-family issues and/or Community issues based on the social ecological model of childhood caries [26]. Quotations that illustrate common themes are used throughout the text.

## 3. Results

### 3.1. Demographics and Dental Status

Eighteen families completed in-depth interviews. Key demographics and dental status characteristics are summarized in Table 1. The children’s mean age was approximately 4 years. Children in the high-caries group averaged 11 decayed teeth and 3 lifetime dental visits, while children in the low-caries group averaged 1 decayed tooth and nearly 7 lifetime dental visits.

As part of the larger study, mothers provided demographic information in the form of a short survey [9]. Mean maternal age was 34 in the high-caries group and 28 in the low-caries group. Generally, mothers averaged 10 years of education. In both groups, mothers had an average of 3–4 children. As much of the study population practices subsistence farming and bartering, socioeconomic status (SES) cannot be accurately assessed by income [23]. For the purpose of describing SES in such a setting, several items were adapted from the material style of life scale (potable water, electricity, cooking gas) [27]. Only half of the families had access to potable water, but most (87% and 94%, respectively) had access to electricity and gas for cooking.

Proximity of the family home to a highway leading into the closest semi-urban areas was a measured descriptor of relative remoteness. Families living within thirty minutes’ walk of the highway were considered to be proximal. The majority of families studied did not live proximally to the highway. Despite the relative remoteness, 80% of families lived within a 5-min walk to a store. Stores consisted of small huts or rooms within homes where mainly processed snacks and drinks, and, more rarely, perishable foods could be purchased.

The small number of subjects limits the power of quantitative comparisons. Nevertheless, some trends can be described: the high-caries families had a higher proportion of male children, mothers with higher education, higher maternal age and evidence of higher socioeconomic status by the availability of gas for cooking, closer location to stores that sold processed food, and half as many dental visits. 

### 3.2. Nutrition

The two most common topics that were discussed in the interviews were nutrition and health. Under each topic, risk factors and protective factors were identified. Figure 1, below, summarizes the main risk and protective factors for ECC that were identified in this study. Common themes included the dramatic nutrition transition over recent decades, from diets based on subsistence-farmed foods to ones based on store-bought packaged goods. Parents frequently discussed the challenge of preserving their healthy indigenous dietary traditions, primarily a low-sugar, plant-based diet. Parents related this to be a particularly difficult struggle in the face of the allure of processed snacks and sugary drinks, and periodic episodes of food scarcity and hunger.

#### 3.2.1. Nutrition Risk Factors

A common theme among high-caries parents was how much diets had changed since their own childhoods. Parents described this change in a context where stores began selling more processed snack foods and sugary drinks; more parents commenced wage-paying jobs outside the home, resulting in a transition from subsistence to cash economy; and both the access to and convenience of processed foods increased. Many parents stated that they never drank soda as children, and tried soda for the first time as adults. Traditionally, children and adults drank *chicha* or herbal tea made from local leaves or grasses boiled in river water or rainwater. By the time they were adults with their own children, they believed that soda was more available, affordable, thirst-quenching, and safer than the local water sources. One father recalled the first time that he gave his son soda:

The first time he tried cola he must have been really small still, because sometimes we would take the little ones into town with us. But we wouldn’t bring enough chicha to last through the day and they’d get thirsty. So we would have to buy them cola and we’d put it in his bottle. I’m not sure how old he was—Maybe six months?

—High-caries parent

In addition to dietary content, parents discussed the change of timing and context of eating, and lamented the loss of parental supervision, nutrition, family cohesiveness and culture that resulted from mothers studying or working outside of the home. Several mothers discussed how their studies or job prevented them from being home to feed their children and the family to eating together, requiring heavier reliance on outside food sources and snacks and less maternal control over children’s diet. One mother said:

I remember when I was a girl, we would eat together as a family. But today, we can’t. I leave early for work and my husband too. My babies leave early for school. Maybe on the weekends we’ll all sit down, but during the work week, we almost never see each other. It’s a total change. On the other hand, for the families that live in the [interior] communities, they don’t have three meals a day... maybe only once a day. But they eat the food from out there [their farms]. Those of us who live closer [to the city], we eat what there is. We rush around and for one reason or another we don’t eat. Since children don’t eat at home, they eat other things outside of the house. So, health is really suffering.

—High-caries parent

Another mother expressed concern that her long hours at work deprived her children of her company, so she tried to make up for the loss by giving them sweets as “treats.”

Maybe because I’m not there, or I’m not paying attention—sometimes due to my work—I try to make it better by giving them treats. I’m not there in the house to be with them and care for them.

—High-caries parent

#### 3.2.2. Nutrition Protective Factors

The low-caries group commonly emphasized their reliance on food harvested from the jungle and their *chacra* (family farm in the jungle). They noted that it was healthier and less expensive than purchasing food from stores in town:

I’m mostly committed to my chacra. Plantain, if I want to buy it in Archidona [the nearby city], it’s so expensive. I can barely buy three or four. I prefer to go to the farm, work, bring back a few plantains, a few bananas, papayas….that way it’s way cheaper. That’s why I’m more committed to the chacra. You know, instead of buying [at the store], I harvest at the chacra. So, with that it’s enough, I mean, I can make a soup with all that.

—Low-caries parent

The low-caries parents also consistently mentioned their efforts to limit their children’s consumption of processed snacks and sugary drinks. They used phrases like “I make them eat [traditional foods]” or “I decide what to buy”, or “I make sure”. They explained how they actively monitored their children’s diets by bringing home fruits and vegetables, prohibiting sweets at home, not giving their children money to buy treats, and avoiding bringing their children into town where they would ask for sweets. One mother described her firm and creative efforts to ensure good nutrition for her children:

I decide what they eat because I know what things are good for them. So sometimes, well I have to lie a bit. I’ve said to my kids “If you don’t eat your food I won’t give you money to buy sweets.” Well, then they eat it all up! Later when they ask me about the money for their sweets I tell them that of course we don’t have money to spend on sweets. I say to them, “But there are apples and mandarins in the fridge. Grab one and leave me alone!”

—Low-caries parent

Other parents described the challenges in setting boundaries around their children’s diet. Some parents described how they learned about the hazards of junk food from their own negative childhood experiences or their observations of children suffering from caries and mouth pain. Some parents explained to their children how junk food would give them a stomachache, diarrhea, or rotten teeth, and believed that these warnings helped to limit their children’s junk food consumption. One mother recounted:

My parents were not educated about these things. So then they also weren’t able to educate us, we would always eat candy, chocolates, popsicles, soda. We had no idea what could damage our teeth. And they didn’t know to tell us that we should brush our teeth. If we wanted to brush, great; if not, that was fine too. So, that’s why my generation, we have such damaged teeth and so many caries, and even mouth pain. It’s taught me, and that’s why now I say “no” to my own kids [about candy]. When my kids have tooth pain, they are so miserable. Sometimes they won’t be able to sleep all night [because of the pain]. So I say “you kids aren’t going to eat candy.” There are rare times that I’ll bring them home sweets. Not every time I go into town like so many of the other moms.

—Low-caries parent

While families mainly discussed the nutritional changes in recent decades in terms of food types and frequency, the factors facilitating these changes were discussed in passing: the social economic status of the family and the economic landscape of the community as a whole. As families and communities transitioned from subsistence farming to wage earning, their access to homegrown produce decreased and their dependence on purchased foods increased.

### 3.3. Health 

Within the topic of health, parents discussed themes around parental monitoring of child health and oral hygiene, and access to medical and dental care.

#### 3.3.1. Health Risk Factors

Some parents discussed the difficulties of negotiation with their children about tooth brushing, handwashing and general hygiene. One mother said:

Sometimes my girls don’t want to brush and I have to make them. They say “No, no, I didn’t eat anything bad. I don’t want to brush.”

—Low-caries parent

Many parents—from both high-caries and low-caries groups—described challenges in accessing dental treatment. Although some families were able to access health care through the public health system, others expressed concerns about the difficulty traveling to clinic sites and experiencing ethnic discrimination at the clinics. One parent said:

The medical attention we get is from the Ministry of Health, and the government says that the care there is equal for everyone, but it’s eye opening. The public [health] employees, supposedly responsible for caring for the public, aren’t supposed to give anyone priority. However, Kichwa people are always given poorer care. In contrast, mestizo people, for them, they attend to them more quickly.

—Low-caries parent

#### 3.3.2. Health Protective Factors

Parents in the low-caries group frequently mentioned their role in monitoring their child’s overall health. Some parents observed that their children got diarrhea when they drank soda or ate too much candy, and subsequently began monitoring their diet more closely. Some parents described how they taught their children to wash their hands to avoid disease transmission, and how they ensured that their children brushed their teeth before bedtime. The parent who described above how her girls sometimes resisted brushing their teeth went on to describe her creative and effective response:

So then I say “You have to brush. The bugs [bacteria] are gonna come after you. And my kids will say, “Where are they? Bring me a mirror so I can see if the bugs are there.” So I tell them, “Brush first and then you’ll see. If you don’t, the bugs are gonna go to school to learn with you.” My kids say “Okay fine! I’ll brush so the bugs don’t come after me.”

—Low-caries parent

An additional theme in the low-caries group was the evolving social norm of shame around “bad teeth and bad breath”, and positive self-esteem from having a healthy smile. Parents discussed the shame they would feel if they sent their child to school with rotten or missing teeth. One parent also described how she used social shame to convince her school-aged children to brush every morning to prevent bad breath and prevent other children at school from avoiding them or teasing them. She said:

If you have bad teeth it can get to the point where you’re embarrassed to even talk because of the bad state of your teeth and because of bad breath, you know? Having good teeth gives one a good self-image as well.

—Low-caries parent

## 4. Discussion

The goal of this study was to gain a better understanding of the complex child, family and community factors underlying the broad disparity in early childhood tooth decay—at both extremes of caries prevalence—in this sample of indigenous children.

Through this study, we gained the opinions of parents, which often get lost when studies focus on quantitative characterizations of a population. All of the parents interviewed were experiencing challenges related to the nutrition transition, with quick access to tasty, low-cost non-nutritious snack foods and beverages. Although parents knew which foods were healthy for their children and which foods were not, many parents found it difficult to put their knowledge into practice. The key practices that families perceived to be integral to maintaining good nutrition and oral health for their children were: (1) owning land and being committed to the tradition of growing harvesting and consuming their own food; and (2) applying effective parenting skills—setting household rules, communicating effectively (using creativity, humor and firmness), guiding and monitoring their children’s behavior, and lovingly say “no” to unhealthy requests. This was especially challenging for town-dwelling parents who relied on store-bought food, and parents who studied or worked outside of the home and lamented that they could not continually supervise their children and often felt urges to compensate by giving their children more unhealthy “treats.”

An intriguing trend seen in this study was that families from *lower* socio-economic backgrounds—including those living in remote locations, relying on farming, and having less formal education—appeared to have children with *better* nutrition and oral health. Although some studies have found better oral health associated with higher income, others have found that in developing countries with significant income inequality, affluence may increase caries risk based on a proportional relationship between sugar consumption (kg/capita/year) and DMFT [27]. These findings suggest that in LMIEs, marginalized subgroups may experience better oral health when they have limited ability to purchase and consume junk food and subsequently rely on traditional dietary practices [28,29]. There may be a hopeful message here for low-income and rural families: ensuring good nutrition and oral health for one’s children does not require high education or high income, but rather the ability to make and enforce healthy choices.

**Figure 2:**
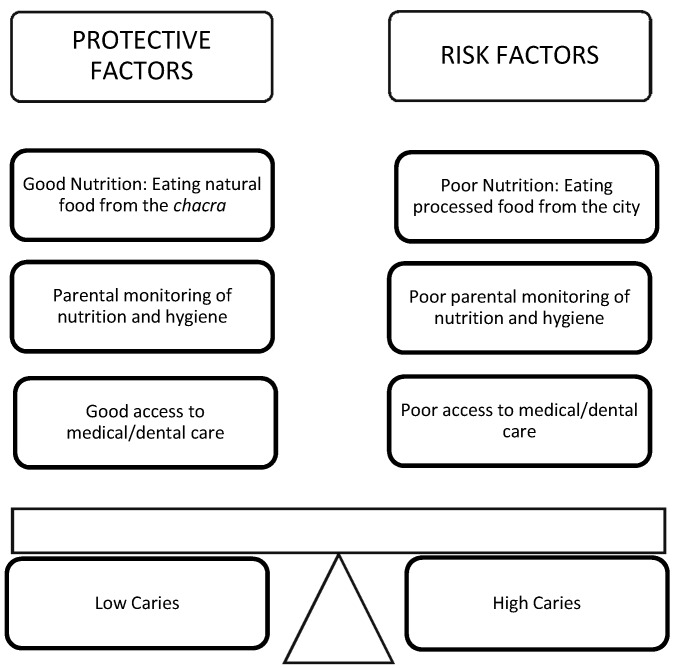
Depiction of the codified protective and risk factors for caries production as conceptualized based on the Caries Balance Model and social ecological children’s oral health disparities conceptual models [28,29].

Considering these findings according to a conceptual model (as depicted in Figure 2), focusing on social-ecological oral health disparities yields implications for interventions at the child-family and community levels. Families need support for land ownership, farming and maintenance of cultural dietary traditions. Beverages like *chicha* and traditional teas could be reincorporated as a better alternative, in moderation, to store-bought sodas in places where fresh water cannot be safely or affordably consumed. For non-land owning families, healthy fruits and vegetables should be made more available in local stores at affordable prices. In addition, families could benefit from programs to promote effective parenting practices consistent with culturally-appropriate values and customs, including providing their children traditional healthy food and limiting junk food consumption, enforcing tooth brushing and other hygiene measures, and ensuring access to non-discriminatory, culturally-sensitive medical and dental care for their family. Exposure to television advertising in the home is a known risk factor, and educational efforts at the family level would be particularly relevant to this population, where so many families have electricity in the home [5,9,10,11].

### 4.1. Implications for Policy and Practice

Communities and regions could develop policies and programs to support families to “make the healthy choice the easy choice”. Health policies and programs should include public works infrastructure to establish a safe, clean, fluoridated and free public water supply to homes, schools and community areas. The Ministry of Education could ensure that schools become health-promotion sites by providing nutrition education, prohibiting junk food from in and around schools, providing nutritious plant-based and low-sugar daily meals and snacks, conducting daily tooth brushing in school, and ensuring child care and afterschool care with good nutrition and oral hygiene for children of working parents. (Notably, at the time of this study, the national preschool and school nutrition program provided children with daily snacks that consisted of sugar cookies, sweet granola bars, and a sweetened beverage.) 

In addition, the Ministry of Agriculture and agricultural colleges could increase promotion of family, school and community gardens, and traditional cultivation and food practices. Local business could be encouraged—potentially with economic incentives—to advertise and sell more locally-grown and nutritious food products, and display the healthier foods at the front of the store (which is typically where junk food is displayed). The Ministry of Health could engage in discussions with the community to identify barriers to care, and utilize indigenous community health workers to help ensure access to discrimination-free medical and dental care, which may be by mobile clinics and telemedicine services for remote communities. Finally, instituting a national tax on sugar-sweetened foods and beverages, as in Mexico, could provide important economic incentives to reduce consumption of non-nutritious foods and beverages, which could be very effective in improving the population’s oral health, nutrition and overall health status [30].

### 4.2. Strengths and Limitations

The findings of this study describe the experience of a small group of Ecuadorian Amazon Kichwa families. They cannot be generalized to a wider population, and do not represent the experience of all Kichwa families, nor all indigenous communities worldwide. While our findings hint at many economic forces at play, our family-focused perspective does not systematically explore all the social, economic and environmental factors influencing families and communities. Yet, this study’s findings increase the understanding of determinants of oral health in an indigenous Amazonian population. Many indigenous populations around the world have experienced similar urbanization and nutrition transitions with higher sugar intake and increased tooth decay, as well as obesity, type 2 diabetes and cardiovascular diseases. This study could provide a framework for future studies of the determinants of oral health in other indigenous communities, and potential interventions for promoting nutrition and oral health globally. 

## 5. Conclusions

The results of this study suggest that the ability to cultivate plant-based foods and apply effective parenting skills may play important roles in the prevention of caries for some indigenous communities. Additionally, low-income families in developing countries may actually receive some protection from caries due to a limited ability to purchase and consume junk food and adherence to traditional dietary practices, while decreased parental involvement and greater access to store-bought foods may increase risk. Future health policies and programs should be aimed at the family and community levels to ensure clean and fluoridated water sources and sugar-free schools, emphasize traditionally cultivated food practices, culturally relevant parenting practices, and access to discrimination-free medical and dental care. Areas of future research include the efficacy of parenting-style education programs and sugar-free school meal programs on child oral health outcomes as well as further investigation of barriers and facilitators of medical and dental care for indigenous communities.

## Figures and Tables

**Table 1:** Child Dental Status and Family Demographics.

Characteristic	High Caries	Low Caries	All
	*n* = 9	*n* = 9	*n* = 18
	Mean (SD) or %	Mean (SD) or %	Mean (SD) or %
Child Characteristics			
Mean age	4.6 (1.3)	3.7 (1.5)	4.1 (1.5)
Male, Female %	67%, 33%	44%, 56%	56%, 44%
Mean # Decayed Teeth (range)	11.4 (1.4) Range = 10–14	1.64 (1) Range = 0–2	6.4 (5.1)
Mean # Child Dental Visits	3	6.8	10
Mother and Household Characteristics			
Mean Maternal Age	34 (8.5)	28 (3.9)	31 (6.8)
Mean # Children	3.8	3.4	5.5
Mean # Years of Education	11.6	8.6	10
% Potable water in home	43%	50%	47%
% Electricity in home	86%	88%	87%
% Cook with gas	100%	88%	94%
Living in community located proximal to highway	12%	11%	12%
Living within 5-min walk to store	86%	75%	80%

## Supplementary Materials

Supplementary File 1
