# Supplementary material for: Maintaining Traditions: A Qualitative Study of Early Childhood Caries Risk and Protective Factors in an Indigenous Community
Source: Int J Environ Res Public Health. 2017 Aug 11;14(8):907. doi: 10.3390/ijerph14080907 (PMC5580610; doi:10.3390/ijerph14080907)
Supplement: Supplementary File 1 [file ijerph-14-00907-s001.pdf]

UNIVERSITY OF CALIFORNIA AT BERKELEY

BERKELEY • DAVIS • IRVINE • LOS ANGELES • MERCED • RIVERSIDE • SAN DIEGO

SAN FRANCISCO • SANTA BARBARA • SANTA CRUZ

**COMMITTEE FOR PROTECTION OF HUMAN SUBJECTS**
**OFFICE FOR THE PROTECTION OF HUMAN SUBJECTS**
University of California, Berkeley
2150 Shattuck Avenue, Suite 313
Berkeley, CA 94704 -5940

**(510) 642-7461**
Fax: (510) 643-6272
Website: http://cphs.berkeley.edu
FWA#00006252

# NOTICE OF APPROVAL FOR HUMAN RESEARCH

| | |
|---|---|
| **DATE:** | *June 07, 2013* |
| **TO:** | *Karen SOKAL-GUTIERREZ, Pub Hlth-JMP* |
| | *Ana Alicia LEVIN, Pub Hlth-JMP, Susan L. IVEY, Pub Hlth-JMP, Diana G S, Diana DARAB, Pub Hlth* |
| **CPHS PROTOCOL NUMBER:** | *2011-04-3178* |
| **CPHS PROTOCOL TITLE:** | *Risk Factors for Early Childhood Caries in Ecuador* |
| **FUNDING SOURCE(S):** | *NONE* |

A(n) *continuing review* application was submitted for the above-referenced protocol. The Committee for the Protection of Human Subjects (CPHS) has reviewed and approved the application on an expedited basis, under Category *4,6,7* of the federal regulations.

Effective Date: *June 07, 2013*
Expiration Date: *June 06, 2014*

*Continuation/Renewal:* Applications for continuation review should be submitted no later than 6 weeks prior to the expiration date of the current approval. *Note: It is the responsibility of the Principal Investigator to submit for renewed approval in a timely manner. If approval expires, all research activity (including data analysis) must cease until re-approval from CPHS has been received.* See Renew (Continue) an Approved Protocol.

*Amendments/Modifications:* Any change in the design, conduct, or key personnel of this research must be approved by the CPHS ***prior*** to implementation. For more information, see Amend/Modify an Approved Protocol.

*Three-year approvals:* Minimal risk, non-federally funded protocols that are not subject to federal oversight may now be given a three-year approval period. Please see Three Year Approvals for information about which protocols can qualify for three-year approvals.

The addition of federal funding or certain modifications that increase the level of risk may require a continuing review form to be submitted and approved in order for the protocol to continue. If one or more of the following changes occur, a Continuing Review application must be submitted and approved in order for the protocol to continue.
• Changes in study procedures that increase risk;
• Addition of federal funds.

*Unanticipated Problems and Adverse Events:* If any study subject experiences an unanticipated problem involving risks to subjects or others, and/or a serious adverse event, the CPHS must be informed *promptly.* For more information on definitions and reporting requirements related to this topic, see Adverse Event and Unanticipated Problem Reporting.

This approval is issued under University of California, Berkeley Federalwide Assurance #00006252.

If you have any questions about this matter, please contact the OPHS staff at 642-7461; fax 643-6272; email ophs@berkeley.edu .

UNIVERSITY OF CALIFORNIA AT BERKELEY

BERKELEY • DAVIS • IRVINE • LOS ANGELES • MERCED • RIVERSIDE • SAN DIEGO SAN FRANCISCO • SANTA BARBARA • SANTA CRUZ

**COMMITTEE FOR PROTECTION OF HUMAN SUBJECTS**
**OFFICE FOR THE PROTECTION OF HUMAN SUBJECTS**
University of California, Berkeley
2150 Shattuck Avenue, Suite 313
Berkeley, CA 94704 -5940

**(510) 642-7461**
Fax: (510) 643-6272
Website: http://cphs.berkeley.edu
FWA#00006252

Sincerely,

Robert B. DI MARTINO
Committee for Protection of Human Subjects
